# The chromatin modifier Satb1 regulates cell fate through Fgf signalling in the early mouse embryo

**DOI:** 10.1242/dev.144139

**Published:** 2017-04-15

**Authors:** Mubeen Goolam, Magdalena Zernicka-Goetz

**Affiliations:** Department of Physiology, Development and Neuroscience, University of Cambridge, Downing Street, Cambridge CB2 3EG, UK

**Keywords:** Satb1, Epiblast, Primitive endoderm, Cell lineage specification, Preimplantation, Mouse

## Abstract

The separation of embryonic from extra-embryonic tissues within the inner cell mass to generate the epiblast (EPI), which will form the new organism, from the primitive endoderm (PE), which will form the yolk sac, is a crucial developmental decision. Here, we identify a chromatin modifier, Satb1, with a distinct role in this decision. Satb1 is differentially expressed within 16-cell-stage embryos, with higher expression levels in the inner cell mass progenitor cells. Depleting Satb1 increases the number of EPI cells at the expense of PE. This phenotype can be rescued by simultaneous depletion of both Satb1 and Satb2, owing to their antagonistic effect on the pluripotency regulator Nanog. Consequently, increasing Satb1 expression leads to differentiation into PE and a decrease in EPI, as a result of the modulation of expression of several pluripotency- and differentiation-related genes by Satb1. Finally, we show that Satb1 is a downstream target of the Fgf signalling pathway, linking chromatin modification and Fgf signalling. Together, these results identify a role for Satb1 in the lineage choice between pluripotency and differentiation and further our understanding of early embryonic lineage segregation.

## Abstract (precis)

**Summary:** Satb1 regulates the cell fate choice between pluripotency and differentiation in the early mouse embryo and is a downstream target of the Fgf signalling pathway.

## INTRODUCTION

The early mammalian embryo must correctly specify three distinct cell lineages: the epiblast (EPI), which gives rise to the embryo proper, and the two extraembryonic lineages, the trophectoderm (TE) and the primitive endoderm (PE), which go on to form crucial supportive structures, the placenta and the yolk sac, respectively. By the 16-cell stage, the mouse embryo has a population of outside and inside cells that follow different fates. The outside cells will give rise to the TE, whereas the inside cells will form the pluripotent inner cell mass (ICM) of the blastocyst. The PE and the EPI are both derived from the ICM of the early blastocyst. Previous research has shown that in the early blastocyst the ICM contains a mixed population of PE and EPI progenitors in a mosaic ‘salt-and-pepper’ distribution, which sort themselves into distinct layers by the time the blastocyst is ready to implant [embryonic day (E) 4.5)] through active cell movements (Chazaud et al., 2006; Kurimoto et al., 2006; Meilhac et al., 2009; Plusa et al., 2008). Even though they are a mixed population early on, the individual cells in the early blastocyst are distinct enough that they go on to form either PE or EPI, but rarely both (Morris et al., 2010). It was shown that EPI precursors expressing the pluripotency marker Nanog secrete Fgf4 ligand in the ICM, which can initiate a signalling cascade in Gata6-positive PE precursors that have the Fgfr2 receptor highly expressed on their membranes (Frankenberg et al., 2011; Kurimoto et al., 2006; Morris et al., 2013; Ohnishi et al., 2014). This Fgf signalling is crucial for preventing Nanog from inhibiting Gata6 and committing cells to a PE cell fate (Frankenberg et al., 2011; Kang et al., 2013; Krawchuk et al., 2013; Schrode et al., 2014). Indeed, when Fgf signalling is inhibited, all ICM cells are directed towards a Nanog-positive EPI cell fate without forming any PE, whereas overexpression results in the opposite phenotype, with all cells being converted into Gata6- and Sox17-positive PE (Chazaud et al., 2006; Feldman et al., 1995; Frankenberg et al., 2011; Nichols et al., 2009; Yamanaka et al., 2010). Although the role of Fgf signalling has been well described in the embryo, much still remains unknown about how the cell-fate choice between PE and EPI occurs. Our aim was to contribute to the identification of new regulators of this lineage decision process.

When we mined a pre-existing data set for genes differentially expressed between the first precursors of ICM (inside cells) and TE (outside cells) at the 16-cell stage (Graham et al., 2014), our attention was drawn to *Satb1*, a chromatin modifier, which was three times more highly expressed in inside cells compared with outside cells, potentially indicating a role within the ICM. Although the role of *Satb1* in the early mouse embryo is unknown, it has been shown to regulate pluripotency in mouse embryonic stem cells (mESCs; Savarese et al., 2009), to regulate self-renewal and pluripotency in both haematopoietic (Will et al., 2013) and trophoblast (Asanoma et al., 2012) stem cells and to promote the differentiation of haematopoietic stem cells (Satoh et al., 2013). Here, we wished to test the hypothesis that *Satb1* contributes to lineage specification within the early mouse embryo.

## RESULTS

### Temporal and spatial expression of Satb1 in preimplantation development

**Fig. 1.**
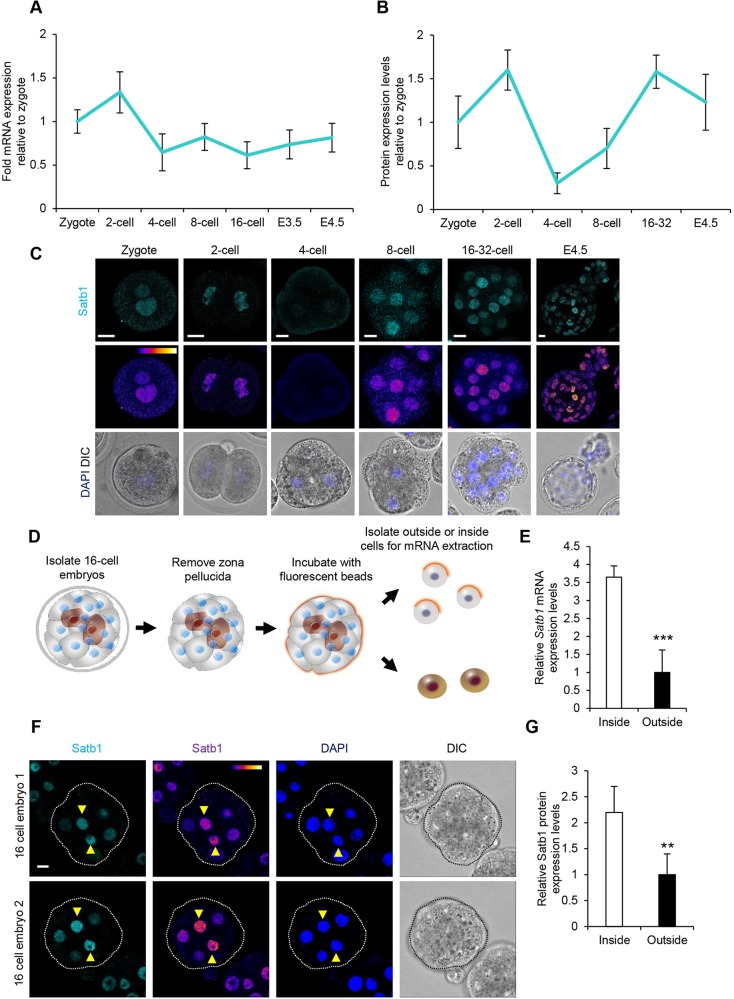
**Satb1** **expression throughout preimplantation development.** (A) qRT-PCR of embryos at zygote (*n*=42), two-cell (*n*=43), four-cell (*n*=39), eight-cell (*n*=41), 16-cell (*n*=41), E3.5 (*n*=54) and E4.5 (*n*=56) to investigate *Satb1* mRNA levels. (B) Quantification of relative fluorescent intensity of Satb1 staining throughout preimplantation development. Representative images are presented in C. (C) Immunofluorescence of Satb1 in zygote (*n*=14), two-cell (*n*=11), four-cell (*n*=12), eight-cell (*n*=15), 16- to 32-cell (*n*=13) and E4.5 (*n*=16) embryos. (D) Scheme of isolation of inside and outside cells at the 16-cell stage for qRT-PCR shown in E. (E) qRT-PCR of inside cells (*n*=35) and outside cells (*n*=41) from 16-cell stage embryos to investigate *Satb1* mRNA levels. (F) Immunofluorescence of Satb1 in 16-cell embryos (*n*=13). Embryo boundary is outlined in white or black. Inside cells are indicated by yellow arrowheads. (G) Quantification of relative fluorescent intensity of Satb1 staining. **P<0.01, ***P<0.001. Representative images are shown in F. Scale bars: 10 μm.

To investigate the potential role of Satb1 in early mouse embryos, we first used qRT-PCR to analyse its expression throughout preimplantation development. This revealed high levels of maternal *Satb1* mRNA at the zygote and two-cell stages, before the zygotic genome is activated, a reduction in *Satb1* at the four-cell stage before expression increased at the eight-cell stage and was fairly stable until the blastocyst stage (Fig. 1A). The presence of maternal mRNA and the stable levels of expression after the eight-cell stage prompted us to investigate Satb1 protein levels by immunofluorescence. We found that the overall expression of protein was highly similar to that of the mRNA, with maternal protein present in the zygote and at the two-cell stage and a drop in expression by the four-cell stage (Fig. 1B,C). Protein levels increased at the eight-cell (in a relatively homogenous fashion; Fig. S1A,B) and 16-cell stages, with Satb1 protein still present until the blastocyst stage in both the TE and ICM (Fig. 1B,C).

We first identified *Satb1* as a gene of interest when examining our earlier mRNA sequencing results (Graham et al., 2014) that revealed it to be three times more highly expressed in inside cells compared with outside cells at the 16-cell stage. To confirm this expression pattern, we determined *Satb1* mRNA levels in inside and outside cells using qRT-PCR. To isolate the individual populations of inside or outside cells, we labelled 16-cell stage embryos by briefly incubating them in a suspension of 0.2 µm fluorescent beads and then segregating inside and outside cells by gentle pipetting, as has been done previously (Graham et al., 2014). Separated individual outside (fluorescent) and inside (non-fluorescent) cells were pooled together for mRNA extraction (Fig. 1D). In total, 35 inside cells and 41 outside cells (over three experiments) were collected. Inside cells were found to have over 3.5 times more *Satb1* mRNA than outside cells (Fig. 1E; *P*<0.001).

Given that Satb1 protein expression peaked at the 16-cell stage, we next investigated whether the differential expression of *Satb1* mRNA at the 16-cell stage is recapitulated at the protein level. Fluorescence intensity measurements of Satb1 staining for outside cells (those that had at least one domain in contact with the outside of the embryo) were compared with the intensity of inside cells (cells that were entirely surrounded by other cells) relative to 4′,6-diamidino-2-phenylindole (DAPI). Intensity measurements were done on the layer-normalized sections using the ImageJ measure function. We found that inside cells had more than twofold more Satb1 protein than the outside cells (Fig. 1F,G). These results indicate that at both protein and mRNA levels, Satb1 is differentially expressed at the 16-cell stage.

### Depletion of Satb1 increases number of pluripotent cells

**Fig. 2.**
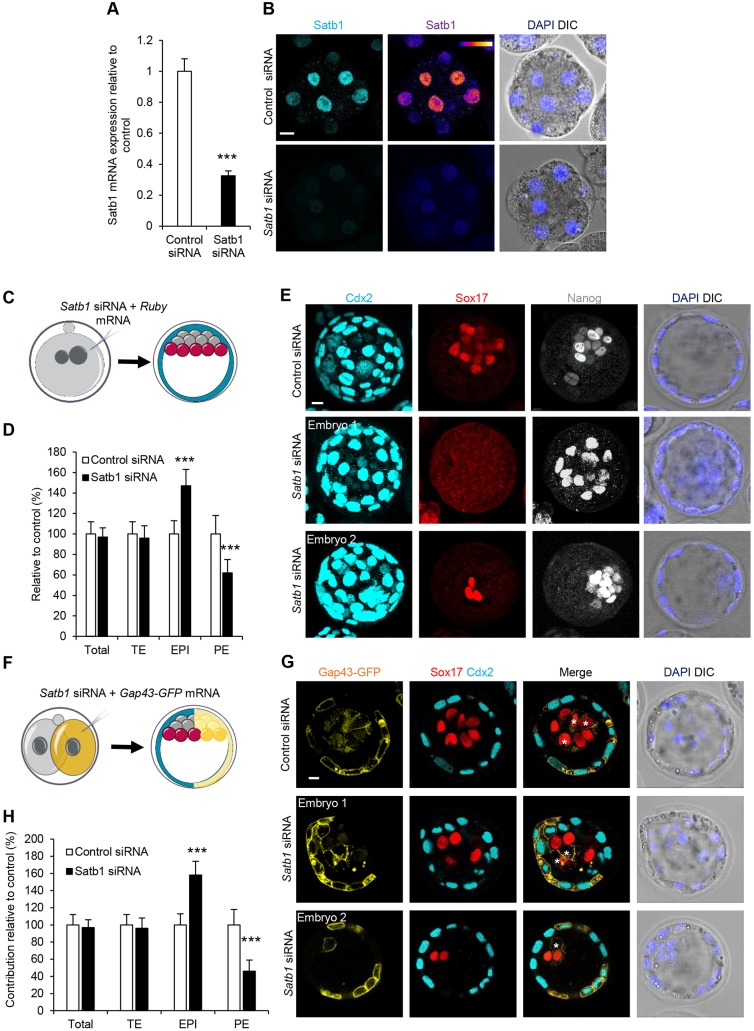
**Reducing Satb1 biases ICM cell fate towards EPI over PE.** (A) qRT-PCR of embryos injected with control siRNA (*n*=52 embryos, three biological repeats) and Satb1 siRNA (*n*=61 embryos, three biological repeats) to investigate *Satb1* mRNA levels. Embryos were injected at zygote and isolated at the eight-cell stage. Embryos injected with Satb1 siRNA show a reduction in *Satb1* mRNA by the eight-cell stage. (B) Immunofluorescence of Satb1 in eight-cell embryos after being injected with control (*n*=11) or Satb1 siRNA (*n*=15). (C) Scheme of Satb1 siRNA experiment shown in D and E. Zygotes were injected with Satb1 siRNA or control siRNA and cultured until E4.5. (D) Contribution of control (*n*=29) and *Satb1* (*n*=36) siRNA-injected embryos to EPI, PE and TE. Representative images of the experiment are shown in E, (E) Confocal images of control and Satb1 siRNA-injected embryos. Nanog, (EPI), Sox17 (PE) and Cdx2 (TE) were used as lineage markers (related to Fig. S2). (F) Scheme of clonal Satb1 siRNA experiment shown in G and H. One blastomere of two-cell stage embryos was injected with Satb1 siRNA, or control siRNA, and *Gap43-*GFP mRNA. Embryos were cultured to the late blastocyst stage, and the contribution of the injected cells' progeny to each lineage was analysed. (G) Confocal images of control (*n*=21, average of 5.2 Sox17-positive/Gap43-GFP-negative and an average of 4.96 Sox17-positive /Gap43-GFP-positive blastomeres per embryo) and *Satb1* (*n*=29, average of 7.84 Sox17-positive/Gap43-GFP-negative and an average of 2.28 Sox17-positive/Gap43-GFP-positive blastomeres per embryo) siRNA-injected embryos. Sox17 (PE) and Cdx2 (TE) were used as lineage markers. Asterisks indicate ICM cells contributed from injected blastomeres. (H) Contribution of Satb1 siRNA-injected cells from experiment shown in G to TE, PE and EPI, relative to control siRNA-injected cells. ***P<0.001. Scale bars: 10 μm.

To determine whether Satb1 might play any role in the preimplantation embryo, we next decreased its expression using a combination of three Satb1-specific small interfering RNAs (siRNAs). We first confirmed that these siRNAs reduced Satb1 at both the mRNA and protein level despite the prevalence of maternal protein and mRNA (Fig. 2A,B) and that the reduction in Satb1 protein persisted until the blastocyst stage (Fig. S1C,D). To test the effect of *Satb1* knockdown, we injected zygotes with *Satb1* siRNA and cultured embryos until the blastocyst stage to compare the cell lineage allocation to embryos injected with a control siRNA (Fig. 2C). We found that Satb1 RNA interference (RNAi) blastocysts had a severely reduced number of PE cells as assessed by Sox17 expression (Fig. 2D,E, Fig. S2). The total number of cells (average of 105 in control and 103 in Satb1 siRNA blastocysts) as well as the number of TE cells (Cdx2^+^ cells; average of 86 in control and 83 in Satb1 siRNA) did not change after Satb1 RNAi (Fig. 2D,E). Importantly, we found that the 38% reduction in PE cells was met with a 47% increase in EPI cells as assessed by the expression of Nanog and Sox17 (Fig. 2D,E), suggesting that reduced levels of Satb1 bias the ICM to produce more EPI rather than PE. To confirm this result, we next injected each Satb1 siRNA individually. We observed the same developmental defect using individual siRNAs as noted with the combination of Satb1 siRNAs, with a reduction in PE cells and an increase in EPI cells (Fig. S1E,F). This phenotype was also found to be proportional to the efficacy of *Satb1* knockdown (Fig. S1G), indicating that the bias in cell fate observed upon *Satb1* depletion is specific to decreased *Satb1* and not attributable to off-target effects. We validated these findings by assessing the expression of two additional PE markers, Gata6 and Pdgfra, as well as an additional EPI marker, Sox2, after Satb1 RNAi, and found a similar bias, with *Satb1*-reduced embryos having an increase in EPI and a decrease in PE by the blastocyst stage (Fig. S3). We next investigated the timing of the effect of Satb1 RNAi in the embryo. We found that at the 16-cell stage (Fig. S4A,B) and at the initiation of the blastocyst, the 32-cell stage (Fig. S4C,D), there was no effect on the distribution or expression pattern of Gata6 or Nanog after Satb1 RNAi. However, by the early blastocyst stage we noted a significant reduction in the number of cells expressing Gata6 after Satb1 RNAi (Fig. S4E,F). These data suggest that although Satb1 has no effect on the initiation of PE specification, it does have a specific role in PE lineage commitment.

The reduction in PE and increase in EPI after Satb1 RNAi suggested that Satb1 could have a role in the cell-fate choice within the ICM. To verify this result, we next determined whether individual blastomeres with reduced Satb1 could have a preferential fate. To this end, we injected one blastomere of two-cell stage embryos with *Satb1* or control siRNA, together with the membrane-bound phosphoprotein *Gap43*-GFP (Benowitz and Routtenberg, 1987) mRNA, which can serve as a marker of cell lineage by labelling the membranes of injected cells (Fig. 2F). The embryos were cultured for 72 h, until the late blastocyst stage, and the contribution to TE, EPI and PE was scored by assessing molecular markers for each lineage and cell position within the embryo. In comparison with control-injected embryos, we found that Satb1 siRNA-injected blastomeres contributed significantly more to the EPI (Fig. 2G,H; *P*<0.001). Consequently, Satb1 siRNA-injected blastomeres also contributed significantly fewer cells to the PE (Fig. 2G,H; *P*<0.001; cells contributed to the PE: 4.96 in control embryos, 2.28 in Satb1 RNAi embryos). In agreement with previous results, injection of Satb1 siRNA into half of the embryo had no effect on the relative total contribution of injected cells or the contribution to the TE when compared with control-injected embryos (Fig. 2G,H). These results indicate that clonal depletion of Satb1 biases cell-fate choice in the embryo: cells with lower Satb1 will preferentially give rise to the EPI as opposed to the PE.

### Increasing Satb1 decreases the number of pluripotent cells

**Fig. 3.**
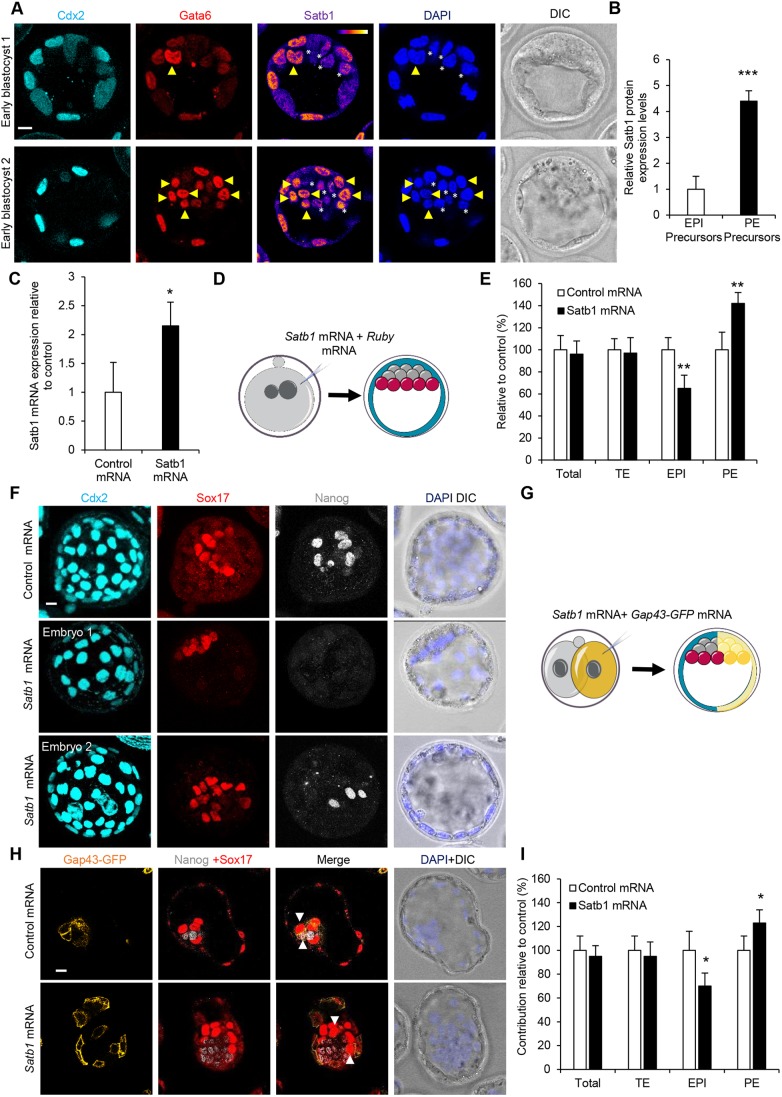
**Effect of Satb1 overexpression on preimplantation development.** (A) Confocal images of Satb1 staining in early blastocysts (*n*=19). Gata6 (PE) and Cdx2 (TE) were used as lineage markers. Yellow arrowheads indicate PE precursors. White asterisks indicate EPI precursors. (B) Quantification of relative fluorescent intensity of Satb1 staining from A. (C) qRT-PCR of embryos injected with control mRNA (*n*=42 embryos) or *Satb1* mRNA (*n*=54 embryos) to investigate *Satb1* mRNA levels. (D) Scheme of *Satb1* overexpression experiment shown in E and F. Zygotes were injected with *Satb1* or control mRNA and cultured until E4.5. (E) Contribution of cells injected with *Satb1* mRNA (*n*=23) to TE, PE and EPI, relative to cells injected with control mRNA (*n*=25). Representative images of the experiment are shown in F. (F) Confocal images of control and *Satb1* mRNA-injected embryos. Nanog (EPI), Sox17 (PE) and Cdx2 (TE) were used as lineage markers. (G) Scheme of clonal *Satb1* mRNA experiment shown in H and I. One blastomere of two-cell stage embryos was injected with *Satb1* mRNA and *Gap43-GFP* mRNA. Embryos were cultured to the late blastocyst stage, and the contribution of the injected cell's progeny to each lineage was analysed. (H) Confocal images of embryos injected with control (*n*=19, average of 5.32 Nanog-positive/Gap43-GFP-negative and an average of 5.16 Nanog-positive/Gap43-GFP-positive blastomeres per embryo) and *Satb1* mRNA (*n*=26, average of 7.27 Nanog-positive/Gap43-GFP-negative and an average of 3.61 Nanog-positive/Gap43-GFP-positive blastomeres per embryo). Sox17 (PE) and Nanog (EPI) were used as lineage markers. White arrowheads indicate ICM cells contributed from injected blastomeres. (I) Contribution of *Satb1* mRNA-injected cells from experiment shown in H to TE, PE and EPI, relative to control mRNA-injected cells. *P<0.05, **P<0.01, ***P<0.001. Scale bars: 10 μm.

Given that reducing Satb1 directs cells towards the pluripotent lineage, we hypothesized that Satb1 might have a role in promoting a PE lineage. To investigate the expression pattern of Satb1 in presumptive PE and EPI cells, we analysed Satb1 expression, together with Gata6 (a marker of PE progenitors), in the blastocyst ICM. We found that Satb1 expression was significantly higher in PE precursors as opposed to EPI precursors (Fig. 3A,B; *P*<0.001), as would be expected of a gene with a role in PE specification. To investigate whether overexpressing *Satb1* might have the opposite effect to its reduction, we reverse transcribed mRNA from a *Satb1* cDNA, injected it into zygotes (400 ng/μl) and let embryos develop until morula stage, when we analysed them by qRT-PCR. We found that the injection of *Satb1* mRNA resulted in a more than twofold increase in *Satb1* mRNA levels (Fig. 3C; *P*<0.05), indicating that overexpressing *Satb1* is effective. To test whether overexpressing *Satb1* mRNA was able to rescue the phenotype seen after Satb1 siRNA, we depleted Satb1 siRNA in the zygote and then overexpressed *Satb1* mRNA in both blastomeres of the two-cell stage embryo (Fig. S5A). Overexpression of *Satb1* was able to return the number of PE and EPI cells to levels similar to controls, providing evidence for the specificity of the siRNA phenotype (Fig. S5B,C).

To test the consequences of increasing *Satb1* mRNA on lineage allocation, we first injected *Satb1* mRNA into zygotes and allowed them to develop until the blastocyst stage and compared the contribution of mRNA-injected embryos to TE, EPI and PE (Fig. 3D). Overexpression of *Satb1* resulted in a significant increase in the number of PE cells (*P*<0.01) and a significant decrease in the number of EPI cells (*P*<0.01) when compared with controls (Fig. 3E,F), the opposite effect of what we found when knocking down *Satb1*. These results indicate that modulating the levels of *Satb1* has a specific effect on the differentiation of the ICM into PE or EPI: an increase in *Satb1* levels pushes ICM cells preferentially to form PE instead of EPI.

To verify this result, we also determined the lineage contribution when increasing Satb1 clonally. To this end, we injected 400 ng/μl of *Satb1* mRNA together with *Gap43-GFP* mRNA into one blastomere of a two-cell embryo, cultured the embryos to the blastocyst stage and assessed lineage contribution using molecular markers as well as cell position (Fig. 3G). We found that *Satb1* mRNA injection resulted in a significant decrease in contribution to the EPI (Fig. 3H,I; *P*<0.05; cells contributed to the EPI: 5.16 in control embryos, 3.61 in Satb1 RNAi embryos) as well as a significant increase in PE contribution relative to control (Fig. 3H,I). These results were not attribuatble to a reduction in total or TE cell contributions, as both *Satb1* mRNA and control mRNA embryos were similar in their TE and total number of cells (Fig. 3H,I). Therefore, clonal overexpression of Satb1 biases ICM cells to form PE and not EPI. Collectively, these results, together with the clonal siRNA results, indicate that modulating the amount of Satb1 in the embryo has a specific effect on cell-fate choice within the ICM.

### Simultaneous depletion of Satb1 and Satb2 rescues Satb1 depletion

**Fig. 4.**
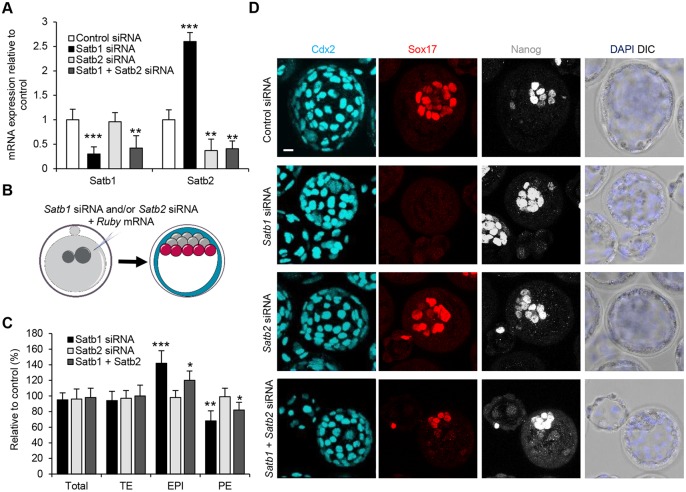
**Rescue of Satb1 siRNA phenotype by Satb2 siRNA.** (A) qRT-PCR of embryos injected with control (*n*=39 embryos), *Satb1* (*n*=52 embryos), *Satb2* (*n*=64 embryos) or Satb1+Satb2 (*n*=52 embryos) siRNA to investigate *Satb1* mRNA levels. (B) Scheme of Satb1 and Satb1 siRNA experiment shown in C and D. Zygotes were injected with Satb1 siRNA and/or Satb2 siRNA and cultured until E4.5. (C) Contribution of cells injected with Satb1 (*n*=16), Satb2 (*n*=29) and Satb1+Satb2 (*n*=32) siRNA to TE, PE and EPI, relative to control [control numbers were normalized to 100, to allow comparison with experimental siRNA-injected embryos (*n*=13) (not shown)]. Representative images of the experiment are shown in D. (D) Confocal images of embryos injected with control, Satb1, Satb and Satb1+Satb2 siRNA. Nanog (EPI), Sox17 (PE) and Cdx2 (TE) were used as lineage markers. *P<0.05, **P<0.01, ***P<0.001. Scale bar: 10 μm.

Satb2 is closely related to Satb1, and it has been shown that knocking down both *Satb1* and *Satb2* simultaneously in mESCs can rescue the impaired differentiation noted in *Satb1*^−/−^ mESCs (Savarese et al., 2009). We therefore decided to investigate whether Satb2 RNAi could also rescue the Satb1 siRNA phenotype in the embryo. We first tested the effectiveness of Satb2 siRNA using qRT-PCR. To this end, Satb1 siRNA, Satb2 siRNA or a combination of both was injected into zygotes at a final total concentration of 12 μM and embryos were collected at the 16-cell stage for mRNA extraction. We found that the knockdown of Satb2 siRNA did not affect *Satb1* mRNA levels but was effective in reducing *Satb2* mRNA by 63% when compared with control (Fig. 4A; *P*<0.01). Interestingly, *Satb1* RNAi resulted in a more than twofold increase in *Satb2* mRNA while reducing *Satb1* mRNA by almost 70% (Fig. 4A; *P*<0.001). The opposite result was found when *Satb1* was overexpressed, with a 50% reduction in *Satb2* mRNA along with a twofold increase in *Satb1* mRNA (Fig. S6; *P*<0.05). These results show that RNAi for both of these closely related genes is specific to each gene and that Satb1 might be a negative regulator of *Satb2*. Additionally, double knockdown of *Satb1* and *Satb2* reduced the levels of both mRNAs to ∼40% of the control values (Fig. 4A; *P*<0.01), indicating that the siRNAs can work simultaneously without interfering with one another when injected into the same embryos.

We next determined the effect of Satb2 siRNA on lineage specification and whether or not it could rescue the Satb1 siRNA phenotype. To this end, Satb1 siRNA, Satb2 siRNA or a combination of both were injected into zygotes at a final total concentration of 12 μM and embryos were allowed to develop until E4.5, when their lineage specification was evaluated (Fig. 4B). We found that Satb2 siRNA by itself had no effect on preimplantation development, with a similar number of TE, PE and EPI cells present compared with controls (Fig. 4C,D). However, double knockdown of *Satb1* and Satb2 siRNA was able to rescue the Satb1 siRNA phenotype significantly, leading to the number of EPI and PE cells being more similar to control embyros (Fig. 4C,D). These results indicate that Satb2 and Satb1 have antagonistic effects on cell-fate choice within the ICM.

### Satb1 modulates the expression of cell-fate regulators

**Fig. 5.**
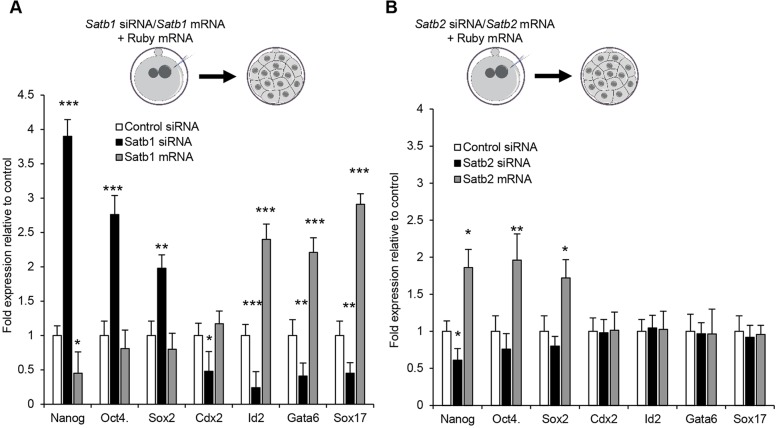
**Gene expression changes after** **modulation of *Satb1* and *Satb2*.** (A) qRT-PCR of various genes in embryos injected with control siRNA (*n*=59 embryos), Satb1 siRNA (*n*=62 embryos) or *Satb1* mRNA (*n*=71 embryos). (B) qRT-PCR of various genes in embryos injected with control siRNA (*n*=59 embryos), Satb2 siRNA (*n*=73 embryos) or *Satb2* mRNA (*n*=58 embryos). In all instances, zygotes were injected and cultured until morula. *P<0.05, **P<0.01, ***P<0.001.

As our results indicate that modulating the levels of Satb1, and to a lesser degree Satb2, affects the cell-fate choice within the ICM, we next wished to determine the changes in gene expression as a result of changing the levels of Satb1 and Satb2, concentrating on key cell-fate determinants at this stage. To this end, we injected Satb1 siRNA, *Satb1* mRNA, Satb2 siRNA or *Satb2* mRNA into zygotes and allowed them to develop until the morula stage (about the 32-cell stage) before mRNA extraction (Fig. 5A,B). We found that Satb1 RNAi resulted in a significant increase in the key EPI regulators *Nanog*, *Oct4* and *Sox2* (Fig. 5A; 3.9-, 2.5- and 1.98-fold, respectively; *Nanog* and *Oct4*: *P*<0.001; *Sox2*: *P*<0.01). It also resulted in a significant decrease in the differentiation markers of the TE, such as *Cdx2* (*P*<0.05) and *Id2* (*P*<0.001), and also PE marker genes, such as *Gata6* (*P*<0.01) and *Sox17* (*P*<0.01). As expected, injection of *Satb1* mRNA had the opposite effect, with a decrease in *Nanog* and increases in *Id2*, *Gata6* and *Sox17* expression (Fig. 5A; *Nanog*: *P*<0.05; *Id2*, *Gata6* and *Sox17*: *P*<0.001). Satb2 RNAi also resulted in decreased expression of *Nanog* (Fig. 5B; *P*<0.05) but did not alter the expression of any of the other genes examined here (Fig. 5B), perhaps accounting for the lack of a phenotype noted after Satb2 RNAi. Interestingly, overexpression of *Satb2* resulted in an almost twofold increase in *Nanog*, *Oct4* and *Sox2* without affecting the other lineage markers (Fig. 5B; *Nanog* and *Sox2*: *P*<0.05; *Oct4*: *P*<0.01), indicating a potential role in promoting an EPI lineage. In agreement with this, we found Satb2 to be co-expressed with Nanog in the early blastocyst and, consequently, to be significantly more highly expressed in EPI cells as opposed to PE cells by the late blastocyst (Fig. S7A,A′,B; *P*<0.001). Overall, these results indicate that Satb1 modulates the expression of cell-fate regulators during preimplantation development and that Satb1 and Satb2 have antagonistic effects on Nanog expression within the early embryo.

### Fgf signalling regulates Satb1

**Fig. 6.**
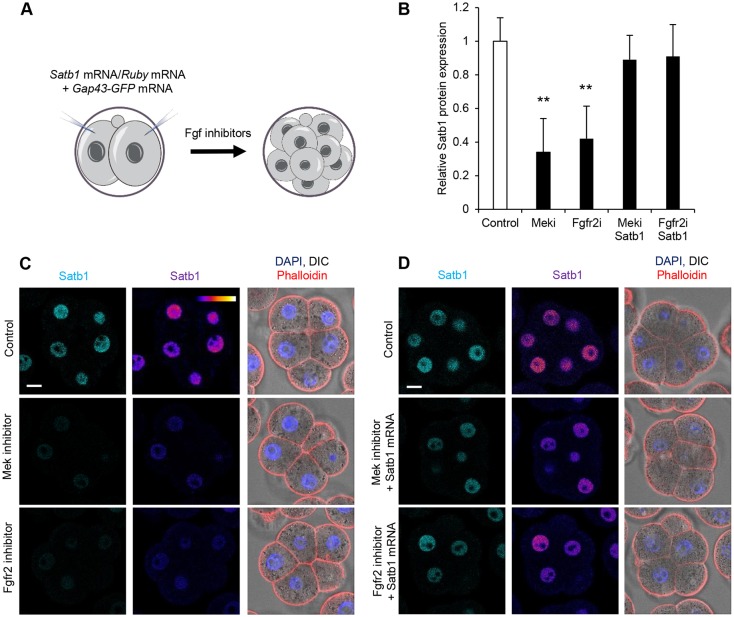
**The effect of Fgf signalling on Satb1 expression.** (A) Scheme of Fgf inhibition experiments in B-D. Two-cell stage embryos were either injected with *Satb1* mRNA or *Ruby* mRNA and treated with a Fgf signalling inhibitor or left untreated before isolation at the eight-cell stage for analysis. (B) Quantification of relative fluorescent intensity of Satb1 staining from C and D. (C) Confocal images of control embryos (*n*=18) and embryos treated with Mek inhibitor (*n*=20), or Fgfr2 inhibitor (*n*=22). (D) Confocal images of control embryos (*n*=18), embryos treated with Mek inhibitor and injected with *Satb1* mRNA (*n*=17) and embryos treated with Fgfr2 inhibitor treated and injected with *Satb1* mRNA (*n*=15). **P<0.01. Scale bars: 10 μm.

As the above results indicated that Satb1 is involved in the specification of PE and EPI in the ICM, we next wished to determine the upstream regulator of *Satb1*. It was shown that the inhibition of Fgf signalling influenced the dependence of mESCs on Satb1 (Savarese et al., 2009). This is particularly interesting because Fgf signalling is crucial to PE fate specification (Frankenberg et al., 2011; Kang et al., 2013; Morris et al., 2013; Yamanaka et al., 2010). To determine whether inhibition of the Fgf signalling pathway affects Satb1 expression in the early mouse embryo, we determined the effects of two different Fgf signalling pathway inhibitors on Satb1 expression. We used an Fgf receptor inhibitor (Morris et al., 2013; Nichols et al., 2009; Yamanaka et al., 2010) and a Mek inhibitor (Nichols et al., 2009; Schrode et al., 2014; Yamanaka et al., 2010) because they are well documented to block PE formation in the embryo. We treated two-cell stage embryos with the Fgf receptor inhibitor at a concentration of 100 nM (Morris et al., 2012) and the Mek inhibitor at a concentration of 0.5 μM (Yamanaka et al., 2010) and then allowed the embryos to develop until the eight-cell stage, when they were fixed and immunostained for Satb1 (Fig. 6A). We found that embryos treated with either inhibitor showed a significant decrease in Satb1 protein by the eight-cell stage (Fig. 6B,C; *P*<0.001).

To examine whether the reduction in Satb1 levels after Fgf inhibition could be rescued by the addition of exogenous *Satb1* mRNA, we injected *Satb1* mRNA into embryos at the two-cell stage and then treated them with inhibitors until the eight-cell stage (Fig. 6A). In these embryos, Satb1 was returned to similar levels as in controls (Fig. 6B,D). These results suggest that Fgf signalling is involved in the regulation of Satb1 in the preimplantation mouse embryo.

## DISCUSSION

The specification of three distinct cell lineages in the mouse embryo occurs during two cell-fate decisions. The first cell-fate decision physically separates the population of ICM and TE cells, whereas the second cell-fate decision further specifies the ICM into the PE and the EPI. It is critical that all three lineages are correctly specified in order to form a blastocyst capable of implanting into the uterine wall and developing further. Here, aiming to identify new regulators that control cell-fate choice in preimplantation development, we discovered the chromatin modifier Satb1 as an important player. Satb1 was first identified in thymocytes, where it is known to regulate gene expression by organizing the structure of higher-order chromatin into loop domains and by acting as a ‘landing platform’ for chromatin-remodelling enzymes (Cai et al., 2006; Yasui et al., 2002). In mESCs, Satb1 was shown to regulate pluripotency by directly repressing Nanog; *Satb1* knockout mESCs maintained Nanog expression even when placed into differentiation medium (Savarese et al., 2009). However, the role of Satb1 in the preimplantation embryo remains unknown. Here, we find that expression of Satb1 is specifically upregulated, at both mRNA and protein levels, in the inner cells of 16-cell stage embryos, when the ICM is first specified, indicating a potential role within the specification of these cells. We further find that Satb1 is specifically upregulated within the PE precursors, signifying its potential importance to the specification of the PE. We confirm this hypothesis by downregulating Satb1, which we show leads to a reduction in the number of PE cells and an increase in the number of EPI cells by the blastocyst stage. In agreement with this, overexpression of Satb1 has an opposite effect on lineage specification and promotes a PE lineage within the ICM. Our clonal knockdown and overexpression experiments further support these findings as we find that blastomeres with reduced Satb1 preferentially give rise to EPI and those with increased Satb1 preferentially give rise to PE. We find that Satb1 does not have an effect on the 16- to 32-cell stage embryo when PE specification is initiated. Rather, it has a role in the commitment of cells within the blastocyst to the PE lineage. We also find that this change in cell fate is attributable to modulation of the expression of a series of lineage-specific genes downstream of Fgf signalling.

We find that although modulating Satb1 expression clearly affects cell-fate commitment in the preimplantation mouse embryo, it rarely results in a complete ablation of either the PE or EPI lineages. This is important when viewed in context of the *Satb1* knockout mice, which survive during embryogenesis but die by 3 weeks of age (Alvarez et al., 2000). If Satb1 is important for the regulation of a balance between pluripotency and differentiation as has been shown here, how can this be reconciled with the lack of a preimplantation phenotype in knockout mice? It has previously been shown that the minimal requirement for successful development is three to four pluripotent cells by the time of implantation (Morris et al., 2012; Soriano and Jaenisch, 1986). If *Satb1*^−/−^ embryos did have a phenotype, based on the results from this study, they would most probably have an ICM with high numbers of EPI cells and low numbers of PE cells. Similar phenotypes have been found in *Fgf4*^+/−^ and *Gata6*^+/−^ embryos, and in both cases, embryos were able to recover by E4.5 (Bessonnard et al., 2014; Krawchuk et al., 2013). Therefore, it is possible that although Satb1 helps to organize and specify the correct number of PE and EPI cells within the ICM, it might not be absolutely essential for embryo survival and can be compensated for, in agreement with the highly regulative nature of mammalian development.

Satb1 is closely related to another family member, Satb2, leading us to investigate whether Satb2 might also have a function in preimplantation development. We find that although downregulating Satb2 by itself has no effect on development, depletion of both genes at once is partly able to rescue the Satb1 RNAi phenotype. This is in agreement with the results in mESCs, because knockdown of both *Satb1* and *Satb2* rescued the disruption in differentiation noted in *Satb1*^−/−^ mESCs (Savarese et al., 2009). Our results indicate that this is likely to be because knocking down *Satb2* reduces *Nanog* mRNA, the opposite effect to reducing *Satb1*. In agreement with this, overexpression of *Satb2* is able to increase *Nanog* expression, providing evidence that Satb2 is a positive regulator of *Nanog*. Satb1 and Satb2 therefore have antagonistic effects on *Nanog* expression. Thus, we hypothesize that when Satb1 alone is reduced it releases its repression on both *Nanog* and *Satb2*, and this is enough to bias cell-fate choice towards the EPI. This bias is strengthened by the fact that Satb1 also acts as a positive regulator of PE differentiation factors Sox17 and Gata6. Knocking down both *Satb1* and *Satb2* removes both the repression and activation of *Nanog* expression, with the net effect of normalizing *Nanog* expression levels. The results we present here showing the effect of Satb2 on *Nanog* might help to explain why it has been impossible to derive *Satb2*^−/−^ mESCs (Savarese et al., 2009), because without appropriate expression level of *Nanog*, it would be impossible to derive functional ESC clones.

The effect of Satb2 on *Nanog* expression raises the question of why reducing *Satb2* levels in the embryo did not affect development to the same degree as modulation of *Satb1*. One explanation is that although Satb2 siRNA was able to reduce *Nanog* levels, more than 65% of *Nanog* mRNA was still present after RNAi. Although Nanog is a crucial factor in determining cell fate, in the highly regulative mouse embryo a 35% reduction in Nanog levels might not be sufficient to drive cell-fate changes. Additionally, our results suggest that although Satb2 might affect only *Nanog* expression in embryos, Satb1 has effects on the expression of multiple genes, including *Cdx2*, *Gata6*, *Id2* and *Sox17*. The same pattern, albeit with the opposite effects on expression, was noted for overexpression of *Satb1* and *Satb2*. The combined effects of Satb1, on numerous genes, are sufficient to drive cell-fate changes within the ICM. Modulating Satb2, however, only moderately affects *Nanog*. This can also explain why the double knockdown of *Satb1* and *Satb2* results in only a partial rescue of the Satb1 siRNA phenotype, because although *Nanog* expression levels might be saved, the effects on the other genes regulated by Satb1 are not.

Finally, our results indicate that Satb1 expression is controlled by Fgf signalling, as we find that inhibition of Fgf signalling inhibits Satb1 expression, which can be restored by the addition of exogenous *Satb1* mRNA. We also attempted to rescue the perturbation in cell fate that is noted after Fgf signalling inhibition (Nichols et al., 2009; Schrode et al., 2014; Yamanaka et al., 2010) by overexpressing *Satb1* mRNA but were unable to restore the expression of PE markers by the blastocyst stage (data not shown). We predict that this is because the Fgf signalling pathway has a wide variety of targets in the mouse embryo, and so rescuing one downstream pathway is not sufficient to overcome the multiple effects of inhibiting Fgf signalling. In agreement, repression of Fgf signalling results in a similar but much stronger phenotype compared with downregulation of *Satb1*, with all ICM cells expressing EPI lineage markers (Frankenberg et al., 2011; Yamanaka et al., 2010). This indicates that Satb1 might be one of the links between Fgf signalling and its downstream targets in early mouse embryos. In agreement with this, Fgf signalling and Satb1 both promote stem cell maintenance and proliferation and inhibit differentiation of trophoblast stem cells (Asanoma et al., 2012; Tanaka et al., 1998).

Taken together, our results suggest that Satb1 might act as a chromatin modifier, modulating gene expression downstream of Fgf signalling, pointing to a crucial missing step of chromatin remodelling that can serve to establish the progenitors of the two distinct lineages within the ICM. We speculate that Satb1 could potentially act in two manners to direct ICM cell fate. Firstly, as Satb1 has been found, through co-immunoprecipitation experiments, to bind directly to the 5′ flanking sequence of *Nanog* in mESCs (Savarese et al., 2009), we predict that Satb1 would also bind directly upstream of *Nanog* to repress its transcription in mouse embryos. Secondly, as we find that Satb1 can regulate the expression of numerous genes in the mouse embryo, we predict that this is a function of its ability to act as a ‘landing platform’ that is able to recruit chromatin-remodelling enzymes to activate or repress gene expression, as has been shown previously (Yasui et al., 2002).

**Fig. 7.**
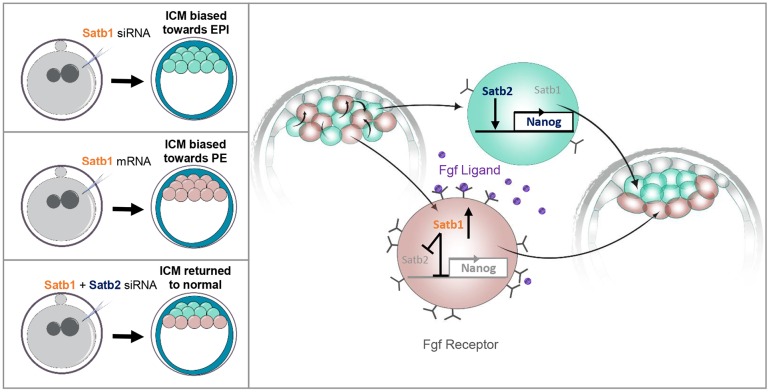
**Model for the role of Satb1 in ICM lineage segregation.** A ‘salt-and-pepper’ distribution of Fgfr2 by the 64-cell stage means that different cells have different responses to Fgf4 signalling. Cells with lower levels of Fgfr2 are less susceptible to Fgf4 signalling and do not upregulate Satb1. Nanog is therefore more highly expressed, promoting the formation of EPI. When *Satb1* is knocked down using a specific siRNA there is an increase in Nanog and therefore an increase in the number of EPI cells present in the embryo. Conversely, cells that are more susceptible to Fgf4 signalling have higher levels of Satb1. Higher expression of Satb1 leads to the inhibition of *Nanog* and *Satb2*. This in turn leads these cells to differentiate into PE. Overexpression of Satb1 with an mRNA is likewise able to bias cell fate towards the PE lineage. Reducing both Satb1 and Satb1 simultaneously is able to restore the balance in PE and EPI by removing both an activator and repressor of *Nanog*.

In conclusion, it can be hypothesized that cells within the ICM have different levels of Fgf receptor, Fgfr2, on their membranes, as has been previously shown (Guo et al., 2010; Krupa et al., 2014; Kurimoto et al., 2006; Morris et al., 2013; Ohnishi et al., 2014). Cells with more receptor are more susceptible to Fgf ligand (which is secreted by the inside cells at this stage) and thus have higher levels of Satb1 (Fig. 7). Higher expression of Satb1 would lead to the inhibition of the pluripotency factors Nanog and Satb2, which in turn would lead them to initiate differentiation into the PE lineage (Fig. 7). Cells that are less susceptible to Fgf4 signalling will have reduced Satb1 which, in turn, leads to more *Nanog* expression and therefore biases cell fate towards the pluripotent EPI lineage. Additionally, loss of both Satb1 and Satb2 removes both an activator and repressor of *Nanog*, resulting in the formation of a normal ICM (Fig. 7). This hypothesis, based on the results we present here, helps to further our understanding of the mechanism that leads to resolution of the ‘salt-and-pepper’ distribution of Gata6- and Nanog-expressing progenitors within the ICM.

## MATERIALS AND METHODS

### Collection of mouse embryos

Embryo recovery was done on superovulated F1 (C57Bl/6xCBA) females between 4 and 6 weeks old, as has been described previously (Piotrowska et al., 2001). Following collection or experimental manipulation, embryos were cultured in drops of potassium-supplemented simplex optimised medium (KSOM; Millipore) supplemented with 4 mg/ml bovine serum albumin under paraffin oil at 37.5°C in air enriched with 5% CO_2_.

### Collection of individual cells

Individual cells for qRT-PCR were collected at the 16-cell stage (78 h after human chorionic gonadotrophin), as has been done previously (Graham et al., 2014). Embryos were isolated directly at the 16-cell stage and incubated in M2 with a fluorescently labelled 0.2 mm microsphere suspension (Polysciences, Inc.) diluted to 1:50 for 30 s. Outside (strongly fluorescent) and inside (non-fluorescent) cells were collected, grouped and placed into Arcturus Biosciences PicoPure RNA isolation kit extraction buffer for mRNA isolation.

### Treatment with chemical inhibitors

To inhibit Fgf signalling, embryos were treated from the zygote to eight-cell stage with an Fgf receptor inhibitor at a concentration of 100 nM (Stemgent; PD173074) or a Mek inhibitor (Stemgent; PD0325901) at a concentration of 0.5 μM, both in KSOM. Inhibitors were dissolved in dimethyl sulphoxide (DMSO; final concentration of DMSO was 0.005%). Control embryos were incubated in the equivalent DMSO concentration but in the absence of the inhibitor. Following treatment with inhibitors, embryos were fixed and processed for immunostaining.

### Microinjection of siRNAs and mRNAs

All microinjections were done with siRNAs for Satb1 and Satb2 as well as AllStars Negative Control siRNA purchased from Qiagen. For siRNA sequences, see Supplementary Materials and Methods. *Satb1* and *Satb2* cDNA (Dharmacon) was cloned into pRN3P as described previously (Zernicka-Goetz et al., 1997). *In vitro* transcription was undertaken on linearized cDNA using the mMessage mMachine T3 kit (Ambion) according to the manufacturer's instructions. Microinjection of embryos with siRNA (always at a final concentration of 12 μM) or mRNA [together with *Ruby* mRNA (200 ng/μl) or *Gap43-GFP* mRNA (400 ng/μl) as markers of injection] was carried out in M2 covered in oil on a glass depression slide using a Femtojet micro-injection system (Eppendorf). Embryos were cultured in KSOM under paraffin oil at 37.5°C in air enriched with 5% CO_2_.

### Immunofluorescence

Immunofluorescence was carried out as described previously (Jedrusik et al., 2008). Multichannel imaging was acquired on a Leica SP5 inverted confocal microscope using Leica LAS AF software and a 20× or 40× oil immersion objective. Confocal *z*-stacks were exported to ImageJ for image processing, intensity measurements and cell counting. For details of the immunofluorescence protocol and intensity measurements as well as antibody details, see supplementary Materials and Methods.

### qRT-PCR

qRT-PCR was carried out as previously described (Goolam et al., 2016). *Gapdh* or *H2afz* was used as the endogenous control. *H2A.Z* was used when different stages of preimplantation development were compared. Three biological repeats were undertaken for every qRT-PCR. For primer details, see supplementary Materials and Methods.

### Statistical analyses

Unless otherwise specified, Student's unpaired *t*-tests were used to test significance (*P<0.05, **P<0.01, ***P<0.001). All error bars represent s.e.m.

## References

[DEV144139C1] Alvarez J. D., Yasui D. H., Niida H., Joh T., Loh D. Y. and Kohwi-Shigematsu T. (2000). The MAR-binding protein SATB1 orchestrates temporal and spatial expression of multiple genes during T-cell development. *Genes Dev.* 14, 521-535. 10716941 PMC316425

[DEV144139C2] Asanoma K., Kubota K., Chakraborty D., Renaud S. J., Wake N., Fukushima K., Soares M. J. and Rumi M. A. K. (2012). SATB homeobox proteins regulate trophoblast stem cell renewal and differentiation. *J. Biol. Chem.* 287, 2257-2268. 10.1074/jbc.M111.287128 22123820 PMC3265903

[DEV144139C3] Benowitz L. I. and Routtenberg A. (1987). A membrane phosphoprotein associated with neural development, axonal regeneration, phospholipid metabolism, and synaptic plasticity. *Trends Neurosci.* 10, 527-532. 10.1016/0166-2236(87)90135-4

[DEV144139C4] Bessonnard S., De Mot L., Gonze D., Barriol M., Dennis C., Goldbeter A., Dupont G. and Chazaud C. (2014). Gata6, Nanog and Erk signaling control cell fate in the inner cell mass through a tristable regulatory network. *Development* 141, 3637-3648. 10.1242/dev.109678 25209243

[DEV144139C5] Cai S., Lee C. C. and Kohwi-Shigematsu T. (2006). SATB1 packages densely looped, transcriptionally active chromatin for coordinated expression of cytokine genes. *Nat. Genet.* 38, 1278-1288. 10.1038/ng1913 17057718

[DEV144139C6] Chazaud C., Yamanaka Y., Pawson T. and Rossant J. (2006). Early lineage segregation between epiblast and primitive endoderm in mouse blastocysts through the Grb2-MAPK pathway. *Dev. Cell* 10, 615-624. 10.1016/j.devcel.2006.02.020 16678776

[DEV144139C7] Feldman B., Poueymirou W., Papaioannou V. E., DeChiara T. M. and Goldfarb M. (1995). Requirement of FGF-4 for postimplantation mouse development. *Science* 267, 246-249. 10.1126/science.7809630 7809630

[DEV144139C8] Frankenberg S., Gerbe F., Bessonnard S., Belville C., Pouchin P., Bardot O. and Chazaud C. (2011). Primitive endoderm differentiates via a three-step mechanism involving Nanog and RTK signaling. *Dev. Cell* 21, 1005-1013. 10.1016/j.devcel.2011.10.019 22172669

[DEV144139C9] Goolam M., Scialdone A., Graham S. J. L., Macaulay I. C., Jedrusik A., Hupalowska A., Voet T., Marioni J. C. and Zernicka-Goetz M. (2016). Heterogeneity in Oct4 and Sox2 targets biases cell fate in 4-cell mouse embryos. *Cell* 165, 61-74. 10.1016/j.cell.2016.01.047 27015307 PMC4819611

[DEV144139C10] Graham S. J. L., Wicher K. B., Jedrusik A., Guo G., Herath W., Robson P. and Zernicka-Goetz M. (2014). BMP signalling regulates the pre-implantation development of extra-embryonic cell lineages in the mouse embryo. *Nat. Commun.* 5, 5667 10.1038/ncomms6667 25514175 PMC4338527

[DEV144139C11] Guo G., Huss M., Tong G. Q., Wang C., Li Sun L., Clarke N. D. and Robson P. (2010). Resolution of cell fate decisions revealed by single-cell gene expression analysis from zygote to blastocyst. *Dev. Cell* 18, 675-685. 10.1016/j.devcel.2010.02.012 20412781

[DEV144139C12] Jedrusik A., Parfitt D.-E., Guo G., Skamagki M., Grabarek J. B., Johnson M. H., Robson P. and Zernicka-Goetz M. (2008). Role of Cdx2 and cell polarity in cell allocation and specification of trophectoderm and inner cell mass in the mouse embryo. *Genes Dev.* 22, 2692-2706. 10.1101/gad.486108 18832072 PMC2559904

[DEV144139C13] Kang M., Piliszek A., Artus J. and Hadjantonakis A.-K. (2013). FGF4 is required for lineage restriction and salt-and-pepper distribution of primitive endoderm factors but not their initial expression in the mouse. *Development* 140, 267-279. 10.1242/dev.084996 23193166 PMC3597205

[DEV144139C14] Krawchuk D., Honma-Yamanaka N., Anani S. and Yamanaka Y. (2013). FGF4 is a limiting factor controlling the proportions of primitive endoderm and epiblast in the ICM of the mouse blastocyst. *Dev. Biol.* 384, 65-71. 10.1016/j.ydbio.2013.09.023 24063807

[DEV144139C15] Krupa M., Mazur E., Szczepańska K., Filimonow K., Maleszewski M. and Suwińska A. (2014). Allocation of inner cells to epiblast vs primitive endoderm in the mouse embryo is biased but not determined by the round of asymmetric divisions (8→16- and 16→32-cells). *Dev. Biol.* 385, 136-148. 10.1016/j.ydbio.2013.09.008 24041854

[DEV144139C16] Kurimoto K., Yabuta Y., Ohinata Y., Ono Y., Uno K. D., Yamada R. G., Ueda H. R. and Saitou M. (2006). An improved single-cell cDNA amplification method for efficient high-density oligonucleotide microarray analysis. *Nucleic Acids Res.* 34, e42 10.1093/nar/gkl050 16547197 PMC1409679

[DEV144139C17] Meilhac S. M., Adams R. J., Morris S. A., Danckaert A., Le Garrec J.-F. and Zernicka-Goetz M. (2009). Active cell movements coupled to positional induction are involved in lineage segregation in the mouse blastocyst. *Dev. Biol.* 331, 210-221. 10.1016/j.ydbio.2009.04.036 19422818 PMC3353123

[DEV144139C18] Morris S. A., Teo R. T. Y., Li H., Robson P., Glover D. M. and Zernicka-Goetz M. (2010). Origin and formation of the first two distinct cell types of the inner cell mass in the mouse embryo. *Proc. Natl. Acad. Sci. USA* 107, 6364-6369. 10.1073/pnas.0915063107 20308546 PMC2852013

[DEV144139C19] Morris S. A., Guo Y. and Zernicka-Goetz M. (2012). Developmental plasticity is bound by pluripotency and the Fgf and Wnt signaling pathways. *Cell Rep.* 2, 756-765. 10.1016/j.celrep.2012.08.029 23041313 PMC3607220

[DEV144139C20] Morris S. A., Graham S. J. L., Jedrusik A. and Zernicka-Goetz M. (2013). The differential response to Fgf signalling in cells internalized at different times influences lineage segregation in preimplantation mouse embryos. *Open Biol.* 3, 130104 10.1098/rsob.130104 24258274 PMC3843820

[DEV144139C21] Nichols J., Silva J., Roode M. and Smith A. (2009). Suppression of Erk signalling promotes ground state pluripotency in the mouse embryo. *Development* 136, 3215-3222. 10.1242/dev.038893 19710168 PMC2739140

[DEV144139C22] Ohnishi Y., Huber W., Tsumura A., Kang M., Xenopoulos P., Kurimoto K., Oleś A. K., Araúzo-Bravo M. J., Saitou M., Hadjantonakis A.-K. et al. (2014). Cell-to-cell expression variability followed by signal reinforcement progressively segregates early mouse lineages. *Nat. Cell Biol.* 16, 27-37. 10.1038/ncb2881 24292013 PMC4062977

[DEV144139C23] Piotrowska K., Wianny F., Pedersen R. A. and Zernicka-Goetz M. (2001). Blastomeres arising from the first cleavage division have distinguishable fates in normal mouse development. *Development* 128, 3739-3748. 11585800 10.1242/dev.128.19.3739

[DEV144139C24] Plusa B., Piliszek A., Frankenberg S., Artus J. and Hadjantonakis A.-K. (2008). Distinct sequential cell behaviours direct primitive endoderm formation in the mouse blastocyst. *Development* 135, 3081-3091. 10.1242/dev.021519 18725515 PMC2768606

[DEV144139C25] Satoh Y., Yokota T., Sudo T., Kondo M., Lai A., Kincade P. W., Kouro T., Iida R., Kokame K., Miyata T. et al. (2013). The Satb1 protein directs hematopoietic stem cell differentiation toward lymphoid lineages. *Immunity* 38, 1105-1115. 10.1016/j.immuni.2013.05.014 23791645 PMC3777575

[DEV144139C26] Savarese F., Dávila A., Nechanitzky R., De La Rosa-Velazquez I., Pereira C. F., Engelke R., Takahashi K., Jenuwein T., Kohwi-Shigematsu T., Fisher A. G. et al. (2009). Satb1 and Satb2 regulate embryonic stem cell differentiation and *Nanog* expression. *Genes Dev.* 23, 2625-2638. 10.1101/gad.1815709 PMC2779756 19933152

[DEV144139C27] Schrode N., Saiz N., Di Talia S. and Hadjantonakis A.-K. (2014). GATA6 levels modulate primitive endoderm cell fate choice and timing in the mouse blastocyst. *Dev. Cell* 29, 454-467. 10.1016/j.devcel.2014.04.011 24835466 PMC4103658

[DEV144139C28] Soriano P. and Jaenisch R. (1986). Retroviruses as probes for mammalian development: allocation of cells to the somatic and germ cell lineages. *Cell* 46, 19-29. 10.1016/0092-8674(86)90856-1 3013418

[DEV144139C29] Tanaka S., Kunath T., Hadjantonakis A. K., Nagy A. and Rossant J. (1998). Promotion of trophoblast stem cell proliferation by FGF4. *Science* 282, 2072-2075. 10.1126/science.282.5396.2072 9851926

[DEV144139C30] Will B., Vogler T. O., Bartholdy B., Garrett-Bakelman F., Mayer J., Barreyro L., Pandolfi A., Todorova T. I., Okoye-Okafor U. C., Stanley R. F. et al. (2013). Satb1 regulates the self-renewal of hematopoietic stem cells by promoting quiescence and repressing differentiation commitment. *Nat. Immunol.* 14, 437-445. 10.1038/ni.2572 23563689 PMC3633104

[DEV144139C31] Yamanaka Y., Lanner F. and Rossant J. (2010). FGF signal-dependent segregation of primitive endoderm and epiblast in the mouse blastocyst. *Development* 137, 715-724. 10.1242/dev.043471 20147376

[DEV144139C32] Yasui D., Miyano M., Cai S., Varga-Weisz P. and Kohwi-Shigematsu T. (2002). SATB1 targets chromatin remodelling to regulate genes over long distances. *Nature* 419, 641-645. 10.1038/nature01084 12374985

[DEV144139C33] Zernicka-Goetz M., Pines J., McLean Hunter S., Dixon J. P., Siemering K. R., Haseloff J. and Evans M. J. (1997). Following cell fate in the living mouse embryo. *Development* 124, 1133-1137. 9102300 10.1242/dev.124.6.1133

